# NLRC4 Mutation in flagellin-derived peptide CBLB502 ligand-binding domain reduces the inflammatory response but not radioprotective activity

**DOI:** 10.1093/jrr/rrz062

**Published:** 2019-10-10

**Authors:** Lili Lai, Ganggang Yang, Xuelian Yao, Lei Wang, Yiqun Zhan, Miao Yu, Ronghua Yin, Changyan Li, Xiaoming Yang, Changhui Ge

**Affiliations:** 1 Graduate School of Anhui Medical University, Hefei 230032, China; 2 Department of Experimental Hematology and Biochemistry, Beijing Institute of Radiation Medicine, Beijing 100850, China; 3 College of Life Science, Henan Normal University; Xinxiang Key Laboratory of Genetic Engineering Medicine, Xinxiang 453731, China; 4 State Key Laboratory of Proteomics, Beijing Proteome Research Center, National Center for Protein Sciences, Beijing Institute of Lifeomics, Beijing 102206, China

**Keywords:** CBLB502, nuclear factor-κB, Toll-like receptor 5, NOD-like receptor protein 4, radioprotection

## Abstract

Bacterial flagellin is a pathogen-associated molecular pattern recognized by surface-localized Toll-like receptor 5 (TLR5) and cytosolic NOD-like receptor protein 4 (NLRC4). CBLB502, derived from *Salmonella* flagellin, exhibits high radioprotective efficacy in mice and primates by regulating TLR5 and the nuclear factor kappa B (NF-κB) signaling pathway. In this study, we examined the effects of CBLB502 and mutations in its NLRC4- and TLR5-binding domains on radioprotective efficacy and the immune inflammatory response. The results showed that CBLB502 mutation with I213A in the TLR5-binding domain significantly reduced NF-κB activity and radioprotective activity, whereas CBLB502 mutation with L292A in NLRC4-binding domain did not. Additionally, CBLB502 with both mutations greatly reduced NF-κB activity and eliminated radioprotection in mice. In contrast, NLRC4-binding domain mutation reduced the secretion of inflammatory interleukin-1β and interleukin-18. CBLB502 exerts its radioprotective effects through both the TLR5 and NLRC4 pathways. Additionally, deletion in the NLRC4-binding domain did not reduce radioprotective activity but reduced the inflammatory response.

## INTRODUCTION

CBLB502, an agonist of Toll-like receptor 5 (TLR5) derived from *Salmonella* flagellin, effectively protects mice and primates from whole-body irradiation and shows low toxicity and immunogenicity [1, 2]. Although antioxidant and scavenging free radical activities and the cytokines granulocyte-colony stimulating factor (G-CSF) and interleukin-6 (IL-6) were reported to be involved in this process [3, 4], the mechanism of radioprotection of CBLB502 remains unclear.

Bacterial flagellin is also recognized by cytosolic NOD-like receptor (NLR) 4 (NLRC4) protein as one of protein pathogen-associated molecular patterns [5]. Flagellin-activated NLRC4 triggers inflammasome assembly, which culminates in caspase-1 activation, interleukin-1β (IL-1β)/IL-18 secretion and cellular pyroptosis [6]. Recently, several studies showed that activation of NLRC4 by flagellin is involved in flagellin-induced and TLR5-mediated immune responses [7], and mutations in the TLR5- and NLRC4-binding domains of flagellin can affect immunity through TLR5 [8], indicating the involvement of NLRC4 in the TLR5-mediated immune response as well as other processes such as radioprotection, which have not been identified.

In this study, we investigated the biological roles of the NLRC4 and TLR5 signaling pathways in CBLB502-mediated radioprotection using CBLB502 mutants within the NLRC4- and TLR5-binding domain and explored the effects of these mutants on the activation, expression and nuclear translocation of nuclear factor (NF)-κB, as well as radioprotective activities and the inflammatory response.

**Fig. 1:**
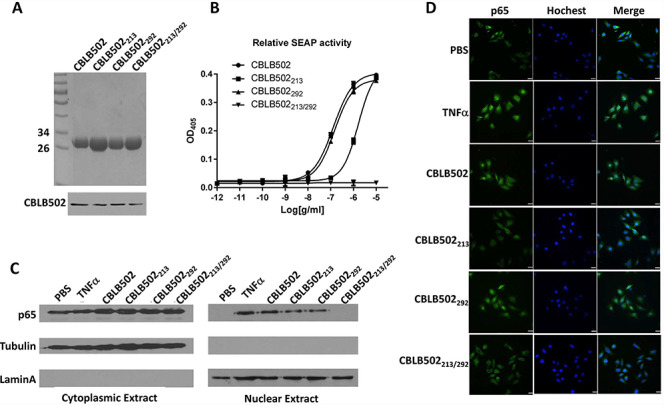
CBLB502 and its mutants affect the expression and translocation of NF-κB. (**A**) SDS-PAGE and western blotting analysis of CBLB502 and its mutant proteins. (**B**) Biological activity of NF-κB according to SEAP reporter assay. (**C**) Expression of NF-κB p65 subunit (p65) in cytosol and nucleus. (**D**) Representative p65 nucleus translocations by CBLB502 and mutant are presented. Scale bar, 25 μm.

**Fig. 2:**
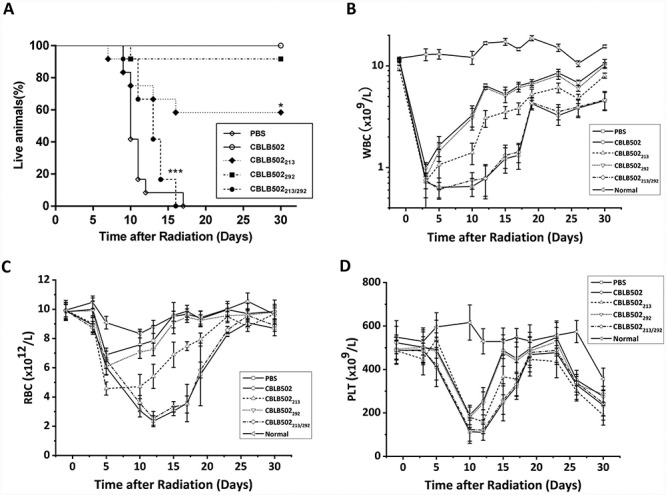
Radioprotective effects of CBLB502 and its mutants. (**A**) Kaplan–Meier survival curves for mice pretreated with CBLB502 and mutants. Peripheral blood counts of (**B**) WBC, (**C**) RBC and (**D**) PLT were analyzed for 30 days. *n* = 10–12. ^*^P < 0.05, ^*^^*^^*^P < 0.001 vs CBLB502.

**Fig. 3:**
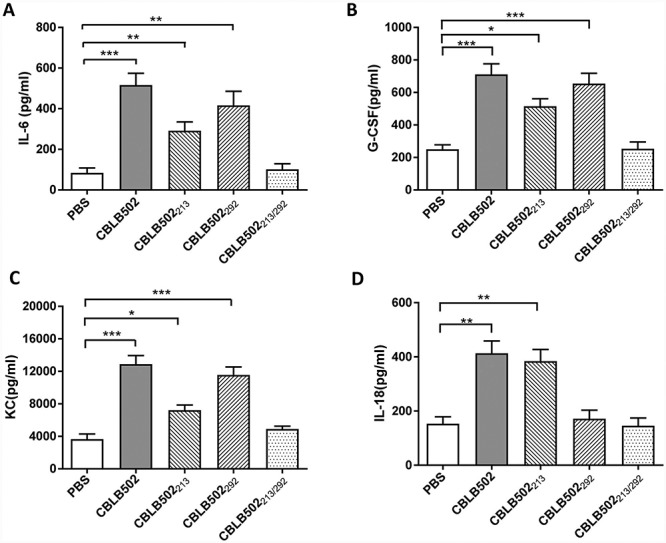
Serum cytokines levels in mice treated with CBLB502 and its mutants. (**A**) IL-6, (**B**) G-CSF, (**C**) KC and (**D**) IL-18. Data are expressed as the means ± SEM. *n* = 5. ^*^P < 0.05, ^*^^*^P < 0.01, ^*^^*^^*^P < 0.001.

## MATERIALS AND METHODS

### Cell culture

The human embryonic kidney cell line HEK293 and human umbilical vein endothelial cells (HUVECs) were cultured at 37°C in 5% CO_2_ in Dulbecco’s modified Eagle’s medium (Gibco/BRL, Grand Island, NY, USA) containing 10% fetal bovine serum (MDgenics, St. Louis, MO, USA).

### Plasmid construction and recombinant protein purification

The plasmid pBV220-CBLB502 was generated by PCR as previously reported [1] and cloned into the temperature-sensitive plasmid pBV220 [9]. The constructs of CBLB502 with the TLR5-binding domain mutation I_213_A (CBLB502_213_), NLRC4-binding domain mutation L_292_A (CBLB502_292_), which are correlated with I_411_ and L_470_ in flagellin [8], or double mutation (CBLB502_213/292_) were generated by site-directed mutagenesis.

CBLB502 and mutant proteins were induced in the *Escherichia coli* BL21 (DE3) strain at 42°C for 6 h after the optical density at 600 nm (OD_600_) of the 30°C bacterial culture reached 0.6–0.8. The cells were sonicated, washed, pelleted and resuspended in 2 M urea overnight. After filtration, the proteins were purified using ion-exchange and hydrophobic-interaction chromatographic purification columns, and eluted in 20 mM Tris-HCl (pH 6.8).

### Mice and radiation

Male C57BL/6 (6–8 week-old) mice were purchased from Vital River Experimental Animal Company (Beijing, China). These mice were maintained under controlled lighting conditions with a 12-h light/12-h dark cycle. All animal experiments were approved by the Institutional Animal Care and Use Committee, Academy of Military Medical Sciences, Beijing, China.

Whole-body irradiation was performed using a ^60^Co γ-ray source (Beijing Institute of Radiation Medicine, Beijing, China). The mice were randomly divided into groups (10–12 mice per group), placed in a ventilated Plexiglas cage and irradiated together. The mice were irradiated with a total dose of 8.0 Gy for survival analysis or 6.5 Gy for peripheral blood analysis at a dose rate of 142 cGy/min.

### SDS-PAGE and western blotting

Proteins were extracted using an NE-PER Nuclear and Cytoplasmic Extraction Reagents kit (Thermo Scientific, Waltham, MA, USA) and subjected to sodium dodecyl sulfate polyacrylamide gel electrophoresis (SDS-PAGE) or western blotting analysis using standard procedures. Primary antibodies were as follows: anti-CBLB502 (Provided by Prof. Haifeng Song, Beijing Institute of Radiation Medicine), anti-p65, anti-Lamin A and anti-tubulin (Santa Cruz Biotechnology, Dallas, TX, USA).

### Luciferase reporter assay

Luciferase reporter assays were performed using an NF-κB secreted alkaline phosphatase (SEAP) reporter assay kit (Novus Biologicals, Littleton, CO, USA) according the manufacturer’s instructions. Briefly, HEK293 cells were cultured in 24-well plates and transfected with pNF-κB/SEAP vectors; 24 h later, CBLB502 was added and the cells cultured for another 12 h, after which alkaline phosphatase activities were measured.

### Immunofluorescence

HUVECs were fixed using 3.5% paraformaldehyde in phosphate buffer saline (PBS). p65 was detected with a rabbit anti-p65 antibody and visualized with a secondary fluorescein isothiocyanate-labeled anti-rabbit antibody. Nuclei were counterstained with Hoechst. Tumor necrosis factor α (TNFα) (R&D Systems, Minneapolis, MN, USA) was used as a positive control. Images were captured under an Olympus IX-71 microscope (Tokyo, Japan).

### Survival evaluation and peripheral blood analysis

Mice were pretreated with 0.2 mg/kg CBLB502 or mutant protein intraperitoneal at 0.5 h pre-radiation; PBS was used as a negative control. After irradiation, the mice were returned to the animal facility and routinely maintained. The survival time was recorded at daily intervals for 30 days. Standard hematological tests to examine white blood cell (WBC), red blood cell (RBC) and platelet (PLT) counts were performed using a hematology analyzer (Celltac E, Nihon Kohden, Tokyo, Japan).

### Cytokine detection

Mice were treated with CBLB502 or mutant protein, and whole blood was collected 2 h later. Serum samples were analyzed by enzyme-linked immunosorbent assay for G-CSF, IL-6, keratinocyte-derived cytokine (KC) (R&D Systems) and IL-18 (Abcam, Cambridge, UK) according to the manufacturers’ protocols.

### Statistical analysis

Statistical analyses were performed using GraphPad Prism 6 software (San Diego, CA, USA). Data were expressed as the mean ± SEM. Statistical significance of survival curves was determined by Kaplan and Meier analysis, and one-way analysis of variance with Dunnett post-test was used to test for differences in cytokine analyses. A value of *P* < 0.05 was considered to indicate significance.

## RESULTS

### CBLB502 mutants reduce activation and nuclear translocation of NF-κB

SDS-PAGE and western blotting results showed purified CBLB502 and mutant proteins as a single 31-kDa band (Fig. 1A). The SEAP reporter assay showed that the activity of the NLRC4-related mutation CBLB502_292_ was similar to that of CBLB502 (EC_50_ of 1.31 × 10^−7^ and 1.49 × 10^−7^, respectively), whereas the TLR5-related mutation CBLB502_213_ showed much lower activity (effective concentration 50 (EC_50_) of 1.65 × 10^−6^). The double mutant CBLB502_213/292_ showed no activity (Fig. 1B).

Furthermore, singly mutated CBLB502 caused lower NF-κB p65 subunit (p65) expression than CBLB502 in the nucleus, but not in the double mutant (Fig. 1C). CBLB502 exhibited similar p65 nuclear translocation to CBLB502_292_ but lower than that of CBLB502_213_; as expected, CBLB502_213/292_ did not translocate p65 into the nucleus (Fig. 1D). These data suggest that the mutations in CBLB502 affected NF-κB expression and translocation, and that both NLRC4 and TLR5 ligand binding are required for CBLB502-mediated NF-κB activation.

### CBLB502 mutation reduces survival after lethal radiation

To test the radioprotective effects of CBLB502 mutants, the proteins were injected into mice 0.5 h before 8.0 Gy radiation. The survival rates of mice injected with the CBLB502_292_ and CBLB502_213_ mutants were 90% and 60%, respectively. All mice pre-treated with CBLB502_213/292_ died by day 16, which is similar to the results in the control group (Fig. 2A). Hemogram analysis indicated that the WBC, RBC and PLT counts in CBLB502_292_ mice were similar to those in CBLB502 mice and greatly decreased in CBLB502_213_ mice, whereas CBLB502_213/292_ mice showed values similar to those in PBS-injected control mice (Fig. 2B–D). These results suggest that the NLRC4-related mutation in CBLB502 slightly decreased the radioprotective effects of CBLB502, whereas the double mutant showed no radioprotective effects.

### CBLB502 NLR mutation reduces inflammatory response

We investigated whether CBLB502 and mutants affected serum cytokine levels. As shown in Fig. 3, all serum cytokines were increased by CBLB502 treatment. However, IL-18 but not IL-6, G-CSF and KC levels were decreased to control levels in CBLB502_292_ mice, whereas IL-6, G-CSF and KC but not IL-18 were decreased to a certain extent by CBLB502_213_. The double mutant did not activate any cytokines. These results indicate that mutation in the NLRC4-binding domain reduced the inflammatory response.

## DISCUSSION

CBLB502 exerts radioprotective effects via NF-κB-mediated cytokines such as G-CSF and IL-6 [3], inhibits radiation-induced apoptosis [2] and scavenges a variety of free radicals [4]. Our data indicate the TLR5-binding domain mutation reduced NF-κB activation by approximately 92%, which agrees with the 95% loss of TLR5 recognition observed in a previous study [10]. However, 40% of the radioprotective effects were retained, which is higher than the remaining cell-based activity. Additionally, NLRC4-binding domain mutation reduced activity and radioprotection by ~10%, whereas double mutation eliminated both cell-based activity and radioprotective effects in mice. These data suggest that the TLR5 pathway is mainly involved and the NLRC4 pathway is partially and subordinately involved in CBLB502-mediated radioprotection.

NLRC4, along with neuronal apoptosis inhibitory proteins, assembles a canonical caspase-1-dependent inflammasome in the cytoplasm that responds to flagellin [11]. Multiple studies have suggested that NLRC4 and TLR5 are both important for innate and adaptive immunity [12]. The promotion of adaptive immunity can be effectively driven by either TLR5-mediated activation of NF-κB or NLRC4-mediated activation of the inflammasome [12]. TLR5 and NLRC4 have collectively redundant roles in lung antibacterial mucosal immunity [6]. Several studies showed that flagellin triggers NLRC4 activation and downregulates TLR5-mediated immune responses [7], or exerts its radioprotection via reactive oxygen species-induced NLRP3 inflammasome-mediated radiation-induced pyroptosis [13].

In agreement with previous reports [7], a mutation in the NLRC4 ligand-binding domain of CBLB502 decreased the secretion of IL-18 but not IL-6, whereas double mutation eliminated the secretion of all cytokines. This result confirms the involvement of NLRC4 in CBLB502-mediated radioprotection, which may be advantageous for further reducing the side-effects of CBLB502.

In summary, our results suggest that CBLB502 exerts its radioprotective effects through both the TLR5 and NLRC4 pathways. TLR5 plays the primary role, whereas NLRC4 plays a subordinate role. Blocking the NLRC4 pathway may reduce the inflammatory response but does not significantly decrease the radioprotective effects. These results may be useful for further decreasing the side-effects of CBLB502 in preventing acute radiation syndrome.
